# Periportal Extramedullary Hematopoiesis With Bile Duct Dilatation: A Case Report of a Rare Condition

**DOI:** 10.7759/cureus.105720

**Published:** 2026-03-23

**Authors:** Marcia Mejia, Sebastian Acevedo Ramirez, Mateo Zapata Naranjo

**Affiliations:** 1 Radiology, Universidad de Antioquia, Medellín, COL; 2 General Medicine, Clínica el Rosario, Medellin, COL

**Keywords:** diagnostic imaging, extramedullary hematopoiesis, magnetic resonance, myeloproliferative neoplasms, primary myelofibrosis

## Abstract

Extramedullary hematopoiesis (EMH) occurs in organs outside the bone marrow. In adults, it is usually linked to inefficient medullary hematopoiesis in the context of hematological diseases. It can manifest as hepatosplenomegaly and masses in different organs that can mimic neoplasms, often requiring histopathological analysis. Integrating clinical and radiological findings is crucial for accurate diagnosis and to avoid unnecessary invasive procedures.

This report presents a case of an adult male patient with EMH and bile duct dilatation (a rare combination) in the context of accelerated phase primary myelofibrosis, whose complications ultimately led to his death despite adequate medical treatment. We emphasize the key radiological features that aid in differentiating EMH from other pathologies.

## Introduction

Extramedullary hematopoiesis (EMH) is the formation of blood cells in organs outside the bone marrow, occurring physiologically or in pathological conditions where medullary hematopoiesis is impaired [1]. While more common in adults with hematological diseases, it can be incidentally found in children due to the persistence of fetal hematopoietic patterns [2]. Its main location is in the paravertebral region of the thorax, spleen, and liver; however, it can affect many other organs, including the lungs, intestine, adrenal glands, skin, and lymph nodes, among others [3]. Symptoms vary depending on the organ affected and the extent of the disease and may include pain and obstructive symptoms [3,4].

Radiologically, EMH typically presents as hepatosplenomegaly and masses. These masses are usually well-defined, exhibit variable density and signal intensity on CT and MRI based on fat content and hematopoietic activity, show minimal or no contrast enhancement, and lack diffusion restriction [3-5]. Periportal involvement is rare, and its association with bile duct dilatation is even less frequently reported [6].

Due to the resemblance of EMH lesions to neoplasms, histopathological confirmation is often pursued. However, the significant risk of bleeding during biopsy underscores the importance of combining radiological findings with clinical data for diagnosis, thereby preventing unnecessary interventions [3-5].

We present a case of EMH with splenomegaly, periportal lesions, and bile duct dilatation in an adult with primary myelofibrosis, highlighting the radiological features that distinguish it from other potential diagnoses.

## Case presentation

A 54-year-old male with primary myelofibrosis, diagnosed six years prior and treated with hydroxyurea and ruxolitinib, presented with five days of diffuse abdominal pain, diarrhea, and fever. Examination revealed tachycardia with otherwise normal vital signs (heart rate 119 bpm, blood pressure 125/76 mmHg, temperature 37 °C, respiratory rate 14 breaths/minute), diffuse abdominal tenderness without peritoneal signs, and splenomegaly. Laboratory tests showed anemia, thrombocytopenia, leukopenia, elevated bilirubin (due to direct bilirubin), and normal transaminases (Table 1).

**Table 1:** Laboratory tests with results and reference values CRP: C-reactive protein, ALT: alanine aminotransferase, AST: aspartate aminotransferase, GGT: gamma-glutamyl transferase

Laboratory tests	Results	Reference values
Hemoglobin	8	14-18 g/dL
Leukocytes	4.2	4.4-12 10^3^/µL
Neutrophils	3.3	1.5-7.26 10^3^/µL
CRP	27	<1 mg/dL
ALT	39	4-42 U/L
AST	32	0-35 U/L
GGT	34	8-38 U/L
Alkaline phosphatase	118	30-120 U/L
Total bilirrubin	2.7	0.3-1 mg/dL
Direct bilirrubin	1.2	0.1-0.3 mg/dL

**Figure 1:**
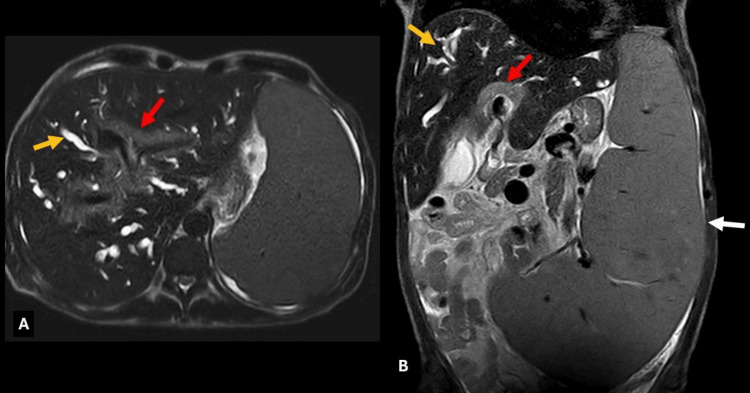
Contrast-enhanced MRI, T2 sequence Axial and coronal acquisitions showing slightly hyperintense periportal soft tissue without thrombosis (red arrows in A and B). There is dilation of the intrahepatic bile duct (yellow arrow in A and B). The coronal sequence shows massive splenomegaly (white arrow in B).

**Figure 2:**
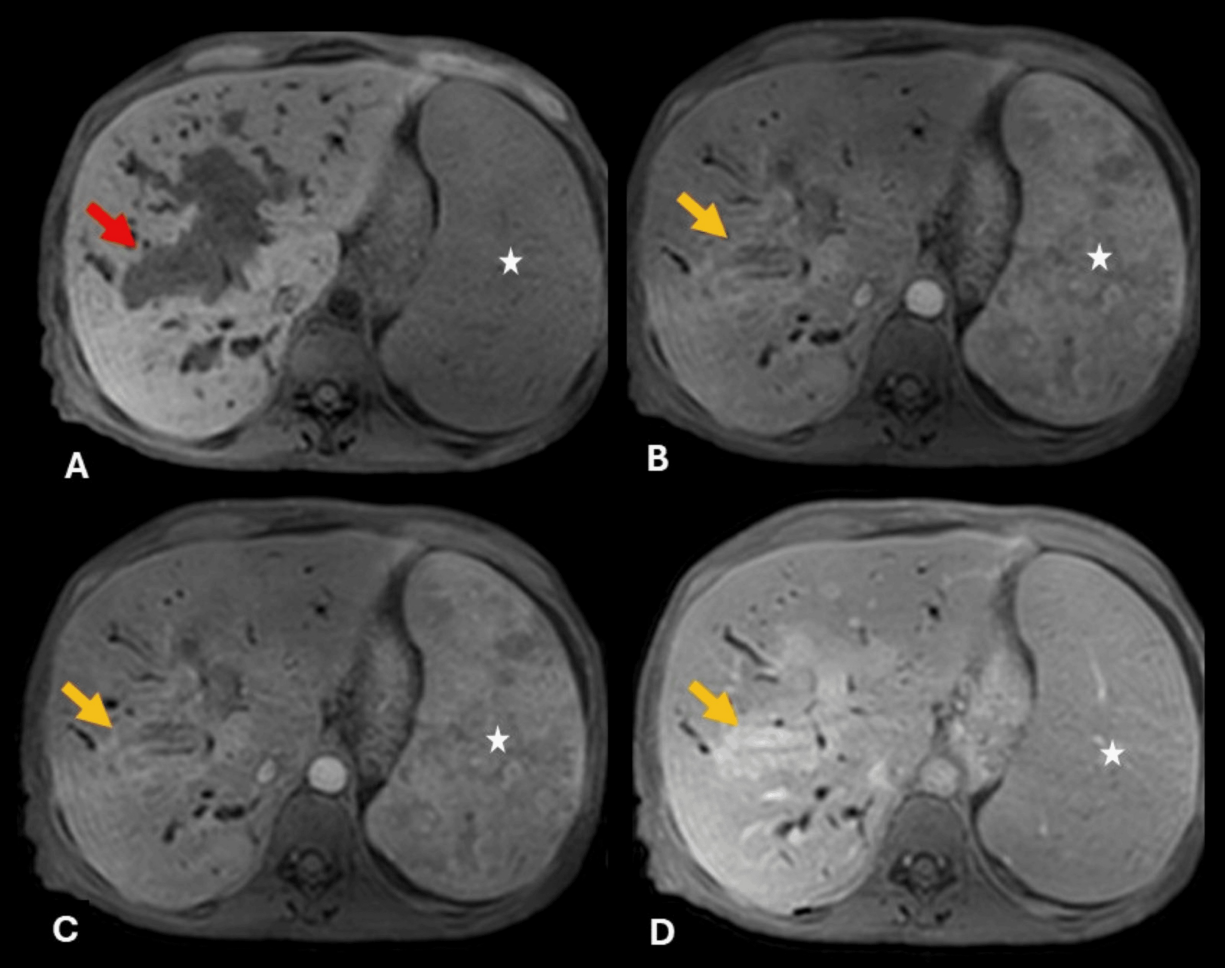
Contrast-enhanced MRI with dynamic T1 sequences reveals nodular periportal soft tissue, hypointense in T1 (red arrow in A), with mild enhancement during the arterial phase that persists without increasing in subsequent phases (yellow arrows). Splenomegaly and heterogeneous splenic enhancement (white star) are also present.

**Figure 3:**
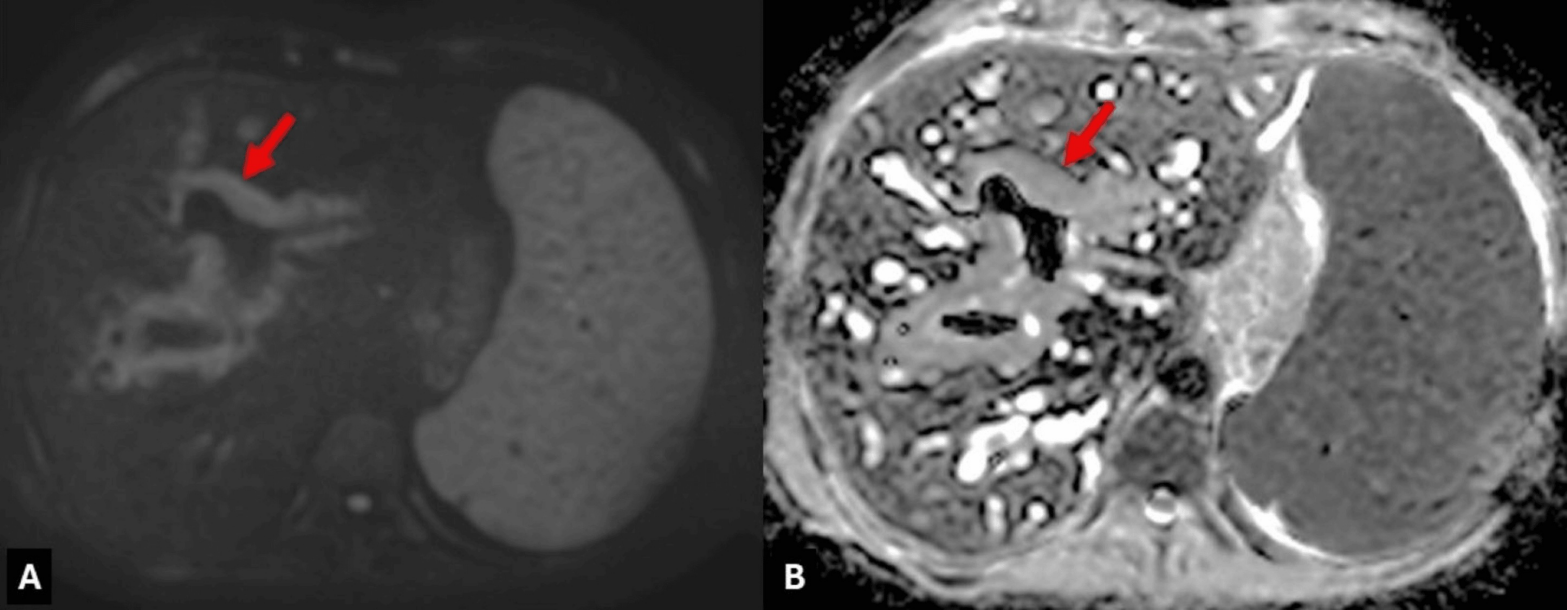
MRI diffusion sequences, B1000 and ADC map (A and B, respectively), show slightly hyperintense nodular periportal soft tissue (red arrows) without diffusion restriction. ADC: apparent diffusion coefficient

**Figure 4:**
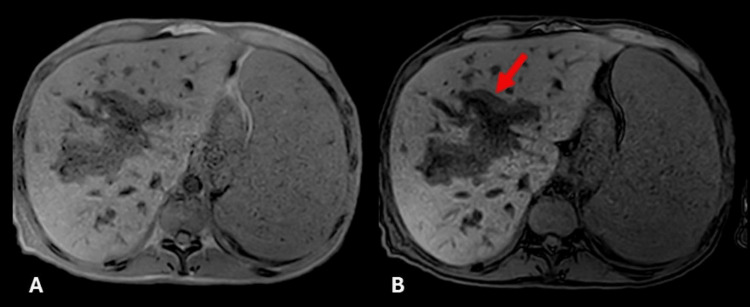
Axial T1 MRI (in/out of phase) shows nodular periportal soft tissue with signal loss on out-of-phase imaging (red arrow in B), consistent with microscopic fat.

*Escherichia coli* (*E. coli)* bacteremia from a gastrointestinal source was also present. Initial ultrasound on the first day of hospitalization showed intrahepatic bile duct dilatation and splenomegaly (images unavailable). For better characterization of the bile duct, a contrast-enhanced MRI was performed on the fourth day of hospitalization, which showed periportal soft tissue with nodular contours, slightly hyperintense on T2 and hypointense on T1, with mild, non-progressive enhancement, no diffusion restriction, and signal drop on out-of-phase sequences, indicating microscopic fat (Figures 1-4).

**Figure 5:**
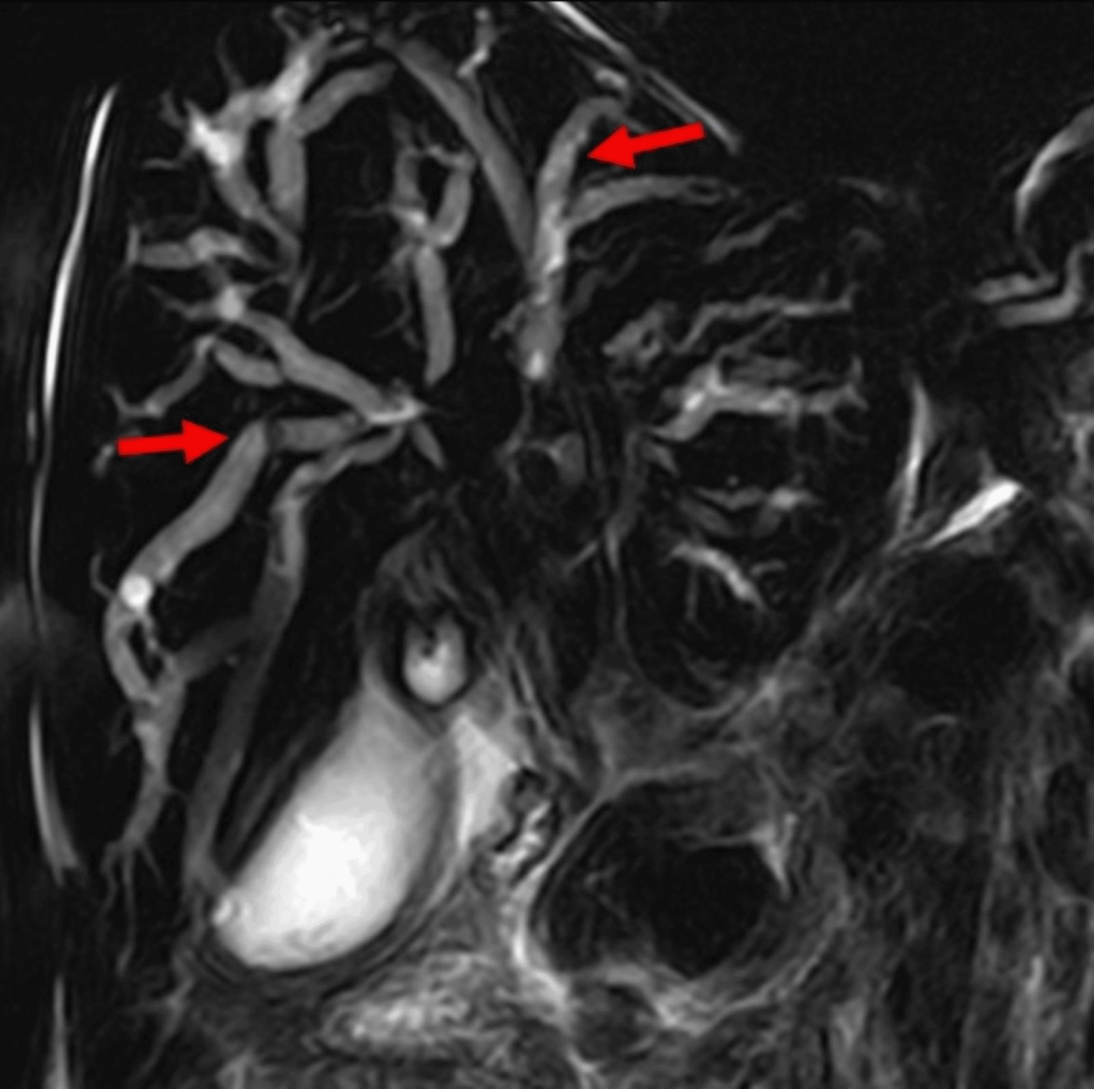
MRCP sequence reveals diffuse, symmetrical intrahepatic bile duct dilation (red arrows). MRCP: magnetic resonance cholangiopancreatography

Symmetrical intrahepatic bile duct dilatation (without identified obstructive lesions) and massive splenomegaly were also observed (Figure 5).

A bone marrow biopsy was performed (images not available), which ruled out progression to leukemia. Based on these findings, accelerated-phase myelofibrosis with EMH involving the spleen and periportal liver was diagnosed. Despite antibiotic (piperacillin/tazobactam) and supportive treatment, the patient developed massive upper gastrointestinal bleeding (secondary to esophageal varices) and died before a planned liver biopsy could be performed. While the biopsy was not obtained, the imaging appearance and clinical context strongly suggested periportal EMH with secondary bile duct involvement.

## Discussion

Hematopoiesis is the process of formation, development, and maturation of blood cells from hematopoietic stem cells. The latter are pluripotent cells whose main location is the bone marrow; however, other organs act as reservoirs of hematopoietic precursor cells, such as the liver, spleen, adipose tissue, small intestine, lungs, and kidneys, among others [1,2]. EMH occurs when hematopoiesis takes place outside the bone marrow, either physiologically (e.g., fetal development and immune response to infections) or in pathological conditions with ineffective medullary hematopoiesis. In pathological conditions where the bone marrow microenvironment is altered, the main site of hematopoiesis changes (especially to the spleen and liver), with migration and expansion of hematopoietic precursors as a compensatory mechanism [1-3].

EMH is associated with various pathologies affecting bone marrow function, such as myelofibrosis, leukemia, lymphoma, and chronic hemolytic anemias (thalassemia, sickle-cell anemia) [1-3]. Primary myelofibrosis (PMF) is a myeloproliferative neoplasm characterized by mutations in hematopoietic stem cells, leading to excessive collagen production and bone marrow fibrosis, resulting in impaired blood cell production and cytopenias. It has a low incidence (~1:100,000), typically affecting individuals in their seventh decade of life, with a slight male predominance and a high mortality rate within 10 years of diagnosis (~80%) [7]. While some patients are asymptomatic, common presentations include malaise, fatigue, fever, pain, paleness, bruising, abdominal pain, and weight loss, among others. This condition is characterized by the appearance of anemia, cytopenias, osteosclerosis (of the axial and appendicular skeleton), and splenomegaly. Its complications include: infections, hemorrhage, portal hypertension, transformation to leukemia/lymphoma, and rarely EMH [7,8].

EMH can occur in almost any organ, most commonly in the paravertebral thorax and intra-abdominal region (hepatosplenic). Paravertebral EMH manifests as well-defined masses isodense to muscle, with mild, homogeneous enhancement and without bone erosion. However, fat or iron content can create a heterogeneous appearance on CT and MRI, with potential hyperintensity on T1 sequences and signal dropout on out-of-phase sequences. Intra-abdominal EMH typically presents as hepatosplenomegaly, often accompanied by splenic infarcts and iron overload (due to repeated transfusions) [2-5]. 

Hepatosplenic involvement can also manifest as masses and mimic neoplasms. The masses have well-defined contours, are isodense to muscle or heterogeneous, and may have minimal or no enhancement on CT images. MRI signal intensity varies based on fat content and hematopoietic activity; they are typically hypointense or isointense on T1-weighted sequences; however, lesions with macroscopic fat content will have hyperintense areas on T1-weighted sequences, and lesions with microscopic fat will have signal loss on out-of-phase sequences. On T2 and STIR, they are usually hypointense, but may be slightly hyperintense (especially if they have fatty content). Enhancement is variable (although often mild), and diffusion restriction is absent (Table 2) [2-4]. 

**Table 2:** Radiological appearance on CT and MRI of EMH with mass appearance EMH: extramedullary hematopoiesis Source: [3-5]

Imaging modalities	Most common findings	Uncommon findings
CT	Well-defined masses, hypo-/isodense, minimal or no enhancement, no bone erosión	Heterogeneous masses, avid enhancement
MRI	Well-defined masses, hypo-/isointense on T1-T2 weighted sequences (may be hyperintense if they have macroscopic fat), signal dropout on out-of-phase sequence (if they have microscopic fat), minimal or no enhancement, no diffusion restriction	Heterogeneous masses, avid enhancement, markedly hyperintense on T1-T2 weighted sequences

Hepatic involvement may also rarely manifest as periportal lesions, with very few cases of this condition reported [6,9]. These lesions are nodular and confluent, surrounding the portal vein branches without causing thrombosis, appearing hypodense with minimal enhancement on CT. On MRI, they are typically hypointense on T1 and T2 images, with little to no enhancement, and without diffusion restriction; although slight hyperintensity and variable enhancement are possible. While periportal and peribiliary involvement is characteristic, bile duct alteration is rarely reported [6,9-11].

Other rare EMH locations include the intraspinal, presacral, nasopharyngeal, paranasal sinus, paratracheal, pleural, cardiac, skin, and urinary tract regions, among others. They manifest as masses with a tomographic and magnetic resonance imaging appearance similar to lesions found in other organs [2-5].

EMH symptoms vary depending on location and size, potentially causing pain, obstructive symptoms, and neurological alterations. Treatment focuses on managing the underlying pathology and may include blood transfusions, splenectomy, and radiotherapy [2,3].

The nonspecific appearance of EMH lesions often necessitates histopathological examination for confirmation. However, given the high risk of bleeding and complications, diagnosis is often based on clinical history and radiological findings, with imaging follow-up [2-5]. 

In our case, differential diagnoses included lymphoma, leukemia (with extramedullary manifestation), metastasis, and cholangiocarcinoma. Lymphoma, while having a predilection for the liver and periportal region, typically shows marked diffusion restriction, which was absent in our case. Extramedullary leukemia rarely involves the liver and also presents with diffusion restriction. Metastases tend to exhibit greater contrast enhancement and diffusion restriction. Intrahepatic cholangiocarcinoma can present with bile duct dilatation but typically demonstrates progressive enhancement and diffusion restriction [2-5]. Additionally, the intralesional fat content in combination with splenomegaly and a history of primary myelofibrosis in our case led to the diagnosis of EMH.

The patient's radiological findings were consistent with rare reports of EMH with periportal involvement but also included bile duct dilatation, which was not attributable to another cause and has not been reported in association with periportal EMH [9-11].

## Conclusions

Extramedullary hematopoiesis is a rare condition usually associated with hematological diseases involving ineffective medullary hematopoiesis. It can manifest as masses compromising the periportal region and mimicking neoplasms. Recognizing key radiological characteristics and integrating them with clinical information is essential for accurate diagnosis and to avoid unnecessary invasive procedures. This case highlights a rare presentation of periportal EMH with bile duct dilatation and splenomegaly in a patient with primary myelofibrosis, where MRI findings, particularly intralesional fat and the absence of diffusion restriction, were crucial for the correct diagnosis.
